# Tyrosine Phosphorylation-Mediated Signaling Pathways in *Dictyostelium*

**DOI:** 10.1155/2011/894351

**Published:** 2011-04-14

**Authors:** Tong Sun, Leung Kim

**Affiliations:** Department of Biological Sciences, Florida International University, Miami, FL 33199, USA

## Abstract

While studies on metazoan cell proliferation, cell differentiation, and cytokine signaling laid the foundation of the current paradigms of tyrosine kinase signaling, similar studies using lower eukaryotes have provided invaluable insight for the understanding of mammalian pathways, such as Wnt and STAT pathways. *Dictyostelium* is one of the leading lower eukaryotic model systems where stress-induced cellular responses, Wnt-like pathways, and STAT-mediated pathways are well investigated. These *Dictyostelium* pathways will be reviewed together with their mammalian counterparts to facilitate the comparative understanding of these variant and noncanonical pathways.

## 1. Introduction

Studies on the tyrosine phosphorylation of *Dictyostelium* were initiated in the early 1990s. Their initial efforts were to identify novel tyrosine phosphatase and kinase genes and categorize their functions using null mutants or overexpressors. 

The earliest reported *Dictyostelium* tyrosine phosphatases were PTP1, PTP2, and PTP3 [1, 2]. A follow-up investigation on these phosphatases showed that PTP1 has a function specific for development at least partly through regulating STATa [3, 4], that PTP2 is regulating MAP Kinase ERK1 [5] and that PTP3 is involved in the STATc regulation [6]. 

DPYK1 and DPYK2 were the earliest *Dictyostelium* tyrosine kinases studied. These kinases were isolated using an antiphosphotyrosine antibody from the cDNA library generated from growth stage cells, and mutant cells lacking DPYK1 displayed defective cell differentiation at the terminal stage of development: spore formation [7]. The second set of kinases, DPYK3, and DPYK4 was cloned, and its recombinant proteins exhibited tyrosine kinase activity *in vitro* [8]. The fifth tyrosine kinase, ZAK1, was isolated from a cDNA expression library generated from differentiated *Dictyostelium* cells with an antiphosphotyrosine antibody [9]. *zak1^−^* cells exhibited severe developmental phenotypes which resembled that of *gsk3^−^* cells, and GSK3 kinase activity was compromised with the concomitant reduction of tyrosine 214 phosphorylation in *zak1^−^* cells. Furthermore, ZAK1 was activated in response to extracellular cAMP treatment in all cells except those lacking cAR3, which is a 7-transmembrane cAMP receptor dominant in the prespore cells [10]. Interestingly, GSK3 was hyperactive in cells lacking cAR4, which is another developmental cAMP receptor prevailing in the prestalk cells [10]. GSK3 also displayed hyperphosphorylation on tyrosine 214 in *car4^−^* cells. A pathway involving tyrosine phosphatase regulating GSK3 tyrosine phosphorylation in response to cAMP was proposed as an antagonistic pathway to the cAR1/ZAK1/GSK3 pathway [11]. Other studies focusing on downstream GSK3 targets identified the *Dictyostelium* beta-catenin homolog Aardvark [12] and STATa [13]. DPYK3 and DPYK4 were revisited in the mid 2000s, and studies showed that DPYK3 has an effect on STAT signaling [14]. The role of DPYK4, which is also known as ZAK2 due to its resemblance to ZAK1, was recently identified as another GSK3 regulator, which activates GSK3 in a cell type-specific mechanism that complements ZAK1 function during development [15]. In contrast to the kinases described above, a receptor-like tyrosine kinase, VSK3, was recently investigated: a null mutant of VSK3 displayed defective phagosome maturation [16]. 

Two decades of study on *Dictyostelium* tyrosine phosphorylation resulted in the detailed mechanisms of how lower eukaryotes regulate GSK3 and STAT utilizing nonreceptor-type tyrosine kinases and phosphatases. Future studies will likely uncover more functions of these enzymes and answer how lower eukaryotes utilize receptor-like tyrosine kinases.

## 2. Tyrosine Kinases in Wnt and Wnt-Like Pathways

The canonical mammalian Wnt pathway controls metazoan development by regulating the level of the key transcription factor, beta-catenin, through a phosphorylation-initiated degradation process. Wnt engaged Frizzled (Fz) receptors regulate beta-catenin stability through multiple signaling components, including Dishevelled (Dsh), Adenomatous Polyposis Coli (APC), Axin, Caseine Kinase 1 (CK1), Glycogen Synthase Kinase 3 (GSK3), PP2A/B56, Presenillin, and beta-catenin, among others. 

**Figure 1:**
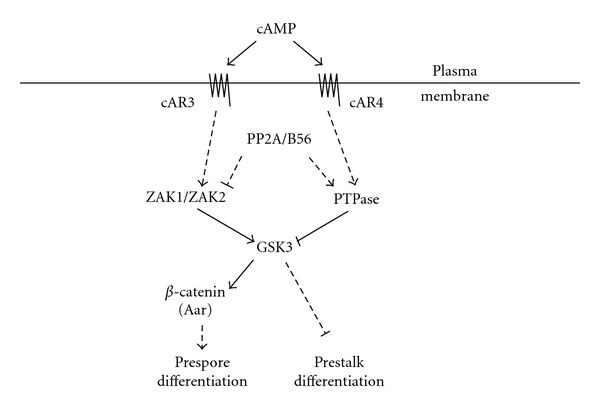
Wnt-like pathways in *Dictyostelium*. During multicellular development, *Dictyostelium *cells first aggregate, then differentiate, into two main types of cells: prespore and prestalk cells. cAMP receptor cAR3 is dominant in prespore cells whereas cAR4 is dominant in prestalk cells. Thus, GSK3 activation prevails in prespore cells through the cAR3/ZAK1/ZAK2 module and is inhibited through the cAR4/PTPase module in prestalk cells. Unlike canonical Wnt signaling in the mammalian system in which GSK3 has a negative effect on beta-catenin, *Dictyostelium* beta-catenin ortholog Aardvark (Aar) is activated by GSK3 and stimulates prespore cell differentiation. B56 subunit of PP2A has an inhibitory effect on GSK3 activity, possibly either by inhibiting ZAK1/2 activities or via stimulating a protein tyrosine phosphatase [17]. Solid lines are supported by direct evidence, while dashed lines are suggested by circumstantial evidence.

Parallel to the effort to delineate the metazoan canonical Wnt pathway, studies attempting to understand *Dictyostelium* development led to the discovery of the Wnt-like pathway outside of the metazoan border. Over decades of effort from multiple research groups resulted in identifying the involvement of signaling components such as ZAK1, ZAK2, GSK3, PP2A/B56, Presenillin, and beta-catenin in the regulation of *Dictyostelium* cell differentiation in response to extracellular cAMP through cAMP receptors 3 (cAR3) and 4 (cAR4) (Figure 1). These pathways activate GSK3 through Tyrosine Kinase Likes (TKLs) ZAK1 and ZAK2 [9, 15] and inhibit GSK3 through an unknown tyrosine kinase [11] and are some of the first and best-studied Wnt-like pathways in the nonmetazoan system. Furthermore, psrA, the *Dictyostelium* homolog of mammalian B56 subunit of protein phosphatase 2A (PP2A), suppressed tyrosine phosphorylation of GSK3 through an unknown mechanism in *Dictyostelium *[17]. A recent realization of the presence of multiple Frizzled like 7-TM receptors in *Dictyostelium* suggested the possibility of another layer of the regulatory network on the Wnt-like pathway [18], but the absence of the Wnt ligand ortholog in the *Dictyostelium* genome contrast the *Dictyostelium* pathway from that of metazoans.

The original discovery of ZAK1-mediated GSK3 activation through tyrosine phosphorylation in the *Dictyostelium* cell was serendipitous. The characterization of ZAK1 function was initially a separate project from that of the interactions between GSK3 and up-stream GPCR receptors (cAR3 and cAR4). However, further analysis on the *zak1^−^* cells exhibited developmental phenotypes, which were very similar to those of *gsk3^−^* and *car3^−^*, yet opposite to those of *car4^−^*. Consistently, GSK3 in developing *zak1^−^* cells displayed weak kinase activity with concomitantly reduced tyrosine 214 phosphorylation. Eventually, these experiments led to the identification of signaling networks regulating GSK3 for cell type specification in *Dictyostelium*. 

Tyrosine kinase mediated GSK3 activation in the mammalian system was initially reported by Woodgett laboratory [19]. Later studies showed that GSK3 could be auto-phosphorylated [20] or phosphorylated by cytoplasmic tyrosine kinases such as Fyn [21]. Further studies demonstrated that the tyrosine kinase Pyk2 could also activate GSK3 in neuronal cells [22–25]. Considering that the majority of these studies used neuronal cells and the importance of Wnt and Wnt-like pathways in neuronal polarity control were recently realized [26, 27], further studies on the role of tyrosine kinase-mediated activation of GSK3 in this field will be significant.

Although the identification of the ZAK1-mediated regulation of the Wnt-like pathway in *Dictyostelium* did not immediately ignite similar discoveries in the mammalian canonical Wnt pathway, the tyrosine kinase, Src, was later found to influence the Wnt-like pathway in *C. elegans* together with receptor tyrosine kinase MES [28]. Furthermore, subsequent studies showed that MET and RON receptor tyrosine kinases induce tyrosine phosphorylation of beta-catenin and activate beta-catenin-mediated transcription in mammalian cells [29, 30]. In addition, MES receptor and Src are involved in the variant Wnt pathway, where Dsh functions positively on GSK3, during *C. elegans* development [28]. Interestingly, other studies revealed that family members of another receptor tyrosine kinase, ROR, not only affect the canonical Wnt pathways [31], but also the variant or noncanonical Wnt pathways, and regulate morphogenesis, cell polarization, and migration [32–37]. The RORs are found to bind to Wnt ligands through its extracellular Frizzled domain (also called cysteine-rich-domain (CRD)). When the expressing cells of ROR2, a family member of ROR, are stimulated with Wnt5a, ROR2 mediates the inhibition of beta-catenin/TCF-mediated transcription. In contrast, the same ligand exhibits the opposite effect on cells expressing Frizzled/LRP [38].

The canonical Wnt pathway may be the first characterized pathway controlled by Wnt ligands, but its name falsely implies that other Wnt-like pathways are of little significance. Given the ever-increasing number of studies demonstrating the interaction between tyrosine kinase and Wnt or Wnt-like signaling pathways, detailed studies of Wnt-like pathways in *Dictyostelium* will be beneficial for the better understanding of the tyrosine phosphorylation-mediated regulation of the Wnt-like pathways in metazoan animals.

## 3. Tyrosine Kinases in STAT Pathways

Signal Transducer and Activator of Transcription (STAT) is a transcription factor whose nuclear localization and control of gene expression are often regulated by growth hormones and cytokines in metazoa. Ligand engaged receptors recruit the cytoplasmic tyrosine kinase, Janus Kinase (JAK), and its subsequent activation leads to the tyrosine phosphorylation of STATs and the activation of target genes, which is called the canonical STAT pathway.

**Figure 2:**
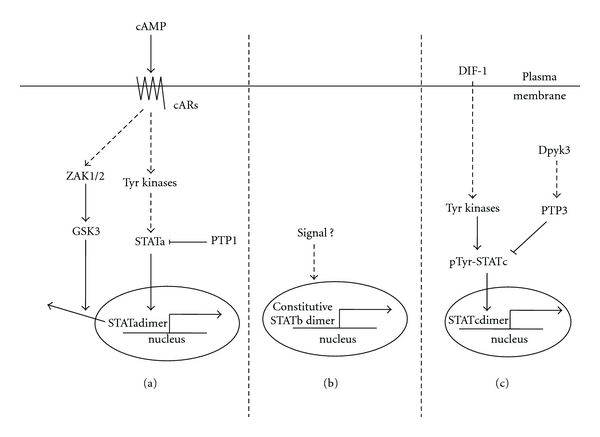
STAT pathways in *Dictyostelium*. (a) cAMP induces activation of a tyrosine kinase which activates STATa, whilst protein tyrosine phosphatase PTP1 acts antagonistically in this process. GSK3, which can be activated by cAMP-ZAK1/2 signaling mentioned earlier, contributes to the nuclear export of STATa. (b) Unlike other STAT proteins, STATb proteins are constitutively localized in the nucleus in a tyrosine phosphorylation independent manner. Elucidation of the mechanism of this unusual STAT protein regulation will be of interest. (c) One of the major prestalk cell morphogens, DIF-1, triggers STATc tyrosine phosphorylation, nuclear localization, and the stimulation of STATc-mediated gene expression. STATc tyrosine phosphorylation is antagonized by protein tyrosine phosphatase PTP3. DPYK3 is known to function positively at the upstream of PTP3 and thus negatively regulate STATc activity [14].

One of the simplest organisms utilizing the STAT pathway is *Dictyostelium*: there are four *Dictyostelium* STAT proteins [39, 40], of which the three better characterized ones will be discussed here. Dd-STATa is activated by cAMP through cyclic AMP receptor 1 (cAR1) and is followed by its nuclear translocation into the prestalk A (pstA) cells of motile *Dictyostelium* slugs (Figure 2(a)) [41]. On the other hand, protein tyrosine phosphatase PTP1 is found to decrease tyrosine phosphorylation of Dd-STATa and inhibit a Dd-STATa-mediated developmental program. Disruption of PTP1 leads to enhanced Dd-STATa signaling [4, 41]. It is reported that Dd-STATa is found to be a substrate of GSK3 in *Dictyostelium*. Upon phosphorylation by activated GSK3, the activation of which is controlled by ZAK1, Dd-STATa is thought to undergo a conformational change that facilitates the binding of exportin/CRM-1 to the nuclear export sequence of Dd-STATa, which promotes nuclear export of STATa [13].

The second STAT protein in *Dictyostelium,* Dd-STATb, an important player in the growth of *Dictyostelium*, is characterized by its unusual SH2 domain, which is characterized by a change of the much conserved arginine to leucine and a 15 amino acid insertion. It is predicted that this feature distinguishes Dd-STATb as a rather unusual functioning mechanism. Unlike Dd-STATa and Dd-STATc whose nuclear localization depends on their tyrosine phosphorylation, Dd-STATb is constitutively dimerized, nuclear localized, and active. The biological function is not impaired even when the predicted tyrosine phosphorylation site is mutated to phenylalanine (Figure 2(b)) [42].

Dd-STATc becomes activated during differentiation or by stress insults. It is reported that STATc is activated by a tyrosine kinase in a cGMP-dependent manner. Overexpression of PTP3 inhibits Differentiation Inducing Factor-1 (DIF-1), or hyperosmotic induced tyrosine phosphorylation of STATc, and thus prevents its nuclear translocation [6, 43]. Although *Dictyostelium* ortholog of mammalian Janus Kinase (JAK) has yet to be identified, detailed studies demonstrate that tyrosine phosphorylation is necessary for the biological function of the STATa and STATc proteins. Cells lacking STATc exhibited compromised development, and reintroduction of the wild-type STATc restored the defects. However, introduction of the mutant STATc, which is incapable of dimerization due to a tyrosine to phenylalanine mutation, failed to rescued the defects [44, 45]. In addition, it is intriguing that one of the *Dictyostelium* tyrosine like kinases, DPYK3, is negatively regulating the STATc pathway: DPYK3 is necessary for the adaptation of Differentiation Inducing Factor-1- (DIF-1-) mediated STATc tyrosine phosphorylation during *Dictyostelium* development (Figure 2(c)) [14]. Considering the unusual as well as the conserved features of *Dictyostelium* STAT signaling pathways, it would not be surprising to encounter variant mammalian STAT pathways reminiscent of certain aspects of *Dictyostelium* STAT pathways. Recent studies indeed uncovered a novel function of metazoan STAT in a noncanonical manner: in *Drosophila*, unphosphorylated STAT associates with a heterochromatin protein, HP1, and regulates the epigenetic gene expression profile [46].

## 4. Tyrosine Kinase in Stress-Induced Pathways

Upon facing a hypertonic environment, *Dictyostelium* cells exhibit adaptive responses to protect themselves. Cell cringing is one such response, which includes remodeling the cortical cytoskeleton through tyrosine phosphorylation of actin and threonine phosphorylation of Myosin II. Although details of the pathway regulating actin tyrosine phosphorylation remain to be determined, more is known about Myosin II regulation: hypertonic stress induced increases in cytosolic cGMP concentration and subsequent activation of Myosin heavy chain kinase triggers the reorganization of Myosin [47]. Interestingly, cGMP also mediates stress-induced STATc activation through a putative STATc tyrosine kinase [48]. A recent study demonstrated that STATc tyrosine phosphorylation is critical for hyperosmotic stress-induced modulation of the global gene expression pattern [49].

The other well-known cellular hypertonic stress response is the alteration of intracellular pH [50]. Given that a single Sodium Hydrogen Exchanger (NHE1) exists in *Dictyostelium* [51], NHE1 is likely involved in the alteration of the intracellular pH. Considering that intracellular pH is also altered by chemoattractants [51], understanding the signaling network controlling intracellular pH would be well worth of the effort.

**Figure 3:**
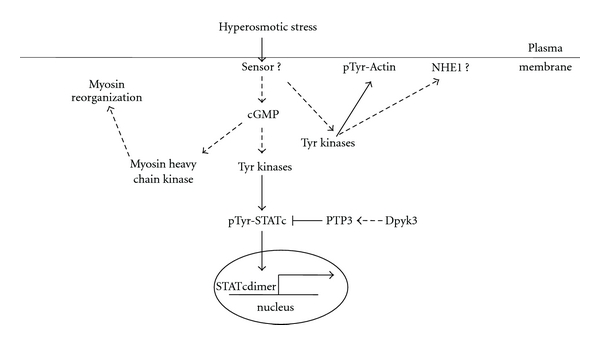
Four hypertonic stress responses in *Dictyostelium*. Cells stressed in hypertonic solution exhibit tyrosine phosphorylation of actin, myosin II heavy chain phosphorylation, activation of a group of genes by STATc, and alteration of intracellular pH. The elevation of the intracellular cGMP level induces activation of the myosin heavy chain kinase and thus remodels the cellular architecture of myosin II organization. The activation of STATc by a Tyrosine Kinase Like is also mediated through increased cytosolic cGMP. The mechanisms of actin tyrosine phosphorylation and intracellular pH alteration need further investigation. Solid arrows are supported by direct evidence, while dashed arrows are suggested by circumstantial evidence.

A suggestion was made from a study using human neutrophils: inhibition of tyrosine kinase prevented hyperosmotic stress-induced NHE1 activation [52]. It will be interesting to determine if tyrosine kinases are involved in the NHE1 activation in *Dictyostelium* cells as well. Therefore, in addition to the Tyrosine Kinase Likes (TKLs) being involved in STAT activation and cortical actin reorganization, a tyrosine kinase may also affect NHE1 function in response to hypertonic stresses (Figure 3).

## 5. Receptor Tyrosine Kinase in Dictyostelium

Although initially characterized *Dictyostelium* Tyrosine Kinase Likes are largely nonreceptor-type kinases, a recently identified VSK3 demonstrated that a receptor tyrosine kinase exists in lower eukaryote such as *Dictyostelium*. Together with VSK1 and VSK2, VSK3 displays typical single-pass transmembrane tyrosine kinase features: a signal sequence, a single transmembrane region (TM), and a Tyrosine Kinase Like domain after the TM. *vsk3^−^* cells were able to internalize phagosomes, but failed to enforce their fusion with late endosomes/lysosomes for the maturation [16, 53]. Identification of VSK3 substrates will be a critical step in understanding the molecular mechanisms of phagocytosis, which will provide novel insight into the host-pathogen interactions where *Dictyostelium* is one of the leading model hosts. It is established that the members of the TAM receptor protein tyrosine kinases (RPTK)—TYRO3, AXL, and MER—have critical roles in phagocytosis of mammalian cells [54, 55]. Given that RPTKs influence their downstream signaling pathways through recruiting SH2 domain containing adaptors on the phosphotyrosine residues on the cytoplasmic region, it is not surprising that TAM receptors also heavily utilize SH2 domain containing signaling molecules [56]. VSK3 likewise contains multiple tyrosine residues at its cytoplasmic region. It is, however, yet to be determined whether activation of VSK3 induces phosphorylation of these tyrosine residues and thus recruits SH2 domain-containing molecules, or rather directly phosphorylates its targets. Further study of VSK3 signaling will definitely contribute to the understanding of the phagocytosis at the molecular level.

## 6. Perspectives

The *Dictyostelium* genome contains 66 Tyrosine Kinase Like proteins based on their sequence motives, which resemble both tyrosine kinases and serine/threonine kinases [39]. Unlike mammalian tyrosine kinases whose members exhibit highly conserved and distinguishing sequence motives, the members of the *Dictyostelium* TKL group display more divergence, and thus their tyrosine kinase activities need to be experimentally confirmed. 

The presence of a total of thirteen SH2 domain-containing proteins in *Dictyostelium* genome [57] also suggests that there are a number of signaling networks mediated by tyrosine phosphorylation. Identification of the binding partners of the SH2 domain proteins and the matching tyrosine kinases and phosphatases will be critical for the holistic understanding of each signaling pathway in each particular cellular context.

As we understand more of the tyrosine kinase-mediated signaling in lower eukaryotes, we will be better equipped with the knowledge of variant and noncanonical pathways, which in turn will facilitate a better selection of a therapeutic target. Furthermore, we will be in a better position to handle various infectious diseases due to the detailed information regarding the process of phagocytosis from lower eukaryotes.
